# Complete Genome Sequences of Four Novel Human Coronavirus OC43 Isolates Associated with Severe Acute Respiratory Infection

**DOI:** 10.1128/genomeA.00452-18

**Published:** 2018-05-24

**Authors:** Darrell L. Dinwiddie, Olga Hardin, Jesse L. Denson, John C. Kincaid, Kurt C. Schwalm, Ashley N. Stoner, Thomas J. Abramo, Tonya M. Thompson, Claire M. Putt, Stephen A. Young, Walter N. Dehority, Joshua L. Kennedy

**Affiliations:** a Department of Pediatrics, University of New Mexico Health Sciences Center, Albuquerque, New Mexico, USA; b Clinical Translational Sciences Center, University of New Mexico Health Sciences Center, Albuquerque, New Mexico, USA; c Department of Internal Medicine, University of Arkansas for Medical Sciences, Little Rock, Arkansas, USA; d Department of Pharmaceutical Sciences, College of Pharmacy, University of New Mexico Health Sciences Center, Albuquerque, New Mexico, USA; e Department of Pediatrics, University of Arkansas for Medical Sciences, Little Rock, Arkansas, USA; f Arkansas Children’s Research Institute, Little Rock, Arkansas, USA; g TriCore Reference Laboratories, Albuquerque, New Mexico, USA

## Abstract

We report here the complete genome sequences of four human coronavirus (HCoV) OC43 isolates generated using targeted viral nucleic acid capture and next-generation sequencing; the isolates were collected in New Mexico and Arkansas, USA, in February (HCoV-OC43/USA/TCNP_0070/2016) and March (HCoV-OC43/USA/ACRI_0052/2016) 2016 and January 2017 (HCoV-OC43/USA/TCNP_00204/2017 and HCoV-OC43/USA/TCNP_00212/2017).

## GENOME ANNOUNCEMENT

Human coronaviruses are enveloped positive-sense single-stranded RNA viruses with the largest genomes of any RNA viruses (1). Coronaviruses cause up to one-third of common colds, with epidemiology peaking in the winter and early spring (2–4). Symptoms can range from subclinical to more severe lower-respiratory-tract involvement in infants (5). There are three groups of coronaviruses which are classified based on genetic and serologic relationships (6). Coronavirus OC43 (HCoV OC43) belongs to the Betacoronavirus genus of the Coronoviridae family.

Nasopharyngeal swabs were collected after consent through studies approved by the institutional review board (IRB) at each institution. An Illumina stranded RNA sequencing library was created from isolated RNA, and hybridization-based enrichment was performed using a respiratory viral panel probe set, which contains 5,683 hybridization probes designed to capture 24 human respiratory viruses (7). Next-generation sequencing was performed on an Illumina MiSeq platform with paired 75-bp reads. Genome sequences were generated by reference-guided alignment to the HCoV OC43 genome (GenBank accession no. AY391777) and *de novo* assembly (CLC Genomics) with manual corrections to any discrepancies from the two methodologies. Annotations were completed using the Genome Annotation Transfer Utility from ViPR (8).

Isolate HCoV-OC43/USA/ACRI_0052/2016 (ACRI_0052) was obtained from a 14-month-old patient who presented to the Arkansas Children’s Hospital emergency department in Little Rock, AR, in March 2016 with a 5-day history of fever, congestion, and wheezing. Isolates HCoV-OC43/USA/TCNP_0070/2016 (TCNP_070), HCoV-OC43/USA/TCNP_00204/2017 (TCNP_00204), and HCoV-OC43/USA/TCNP_00212/2017 (TCNP_00212) were obtained from children presenting to the University of New Mexico Children’s Hospital (UNMCH). TCNP_0070 was isolated from a 9-month-old patient who presented to UNMCH in February 2016 with a 3-day history of fever, cough, and congestion. TCNP_00204 was obtained from a 4-month-old who presented with a 1-day history of cough and congestion to UNMCH in January 2017, was admitted to the hospital, and required an 8-day stay in the pediatric intensive care unit. Isolate TCNP_00212 was obtained from a 5-month-old with preexisting lung disease who presented with a history of 6 days of cough and wheezing and was admitted to UNMCH in January 2017.

Sample ACRI_0052 had 1,191,015 sequencing reads align to the HCoV OC43 RefSeq genome (GenBank accession number AY_391777), which resulted in a mean genome coverage of 2,329×. The genomes of samples TCNP_0070, TCNP_00204, and TCNP_00212 had 293,026, 96,808, and 250,215 aligned sequencing reads, which resulted in mean coverages of 717×, 236×, and 611×, respectively. The genomes of isolates ACRI_0052, TCNP_0070, TCNP_00204, and TCNP_00212 varied from the AY_391777 genome by 346, 334, 550, and 565 nucleotides (nt), of which 137, 150, 99, and 98 were predicted to cause amino acid changes, respectively. In comparison with each other at the genome level, TCNP_00204 and TCNP_00212 from New Mexico were 99.8% identical to each other and 99.5% identical to TCNP_0070, also from New Mexico. Yet, phylogenetic comparison through nearest-neighbor joining revealed that two of the isolates from New Mexico, TCNP_00204 and TCNP_00212, grouped closely to each other and with strains with accession numbers KY369907 and SC9741, whereas the other New Mexican isolate, TCNP_0070, grouped together with ACRI_0052 and was most closely related to the isolates with accession numbers KX538976 and KY967360.

### Accession number(s).

The whole-genome sequences of the isolates have been deposited in GenBank under the accession numbers MF314143 (HCoV-OC43/USA/ACRI_0052/2016), MF374983 (TCNP_0070), MF374984 (TCNP_00204), and MF374985 (TCNP_00212).
